# Mapping of the Thermal Microenvironment for Dairy Cows in an Open Compost-Bedded Pack Barn System with Positive-Pressure Ventilation

**DOI:** 10.3390/ani12162055

**Published:** 2022-08-12

**Authors:** Carlos Eduardo Alves Oliveira, Ilda de Fátima Ferreira Tinôco, Flávio Alves Damasceno, Victor Crespo de Oliveira, Gabriel Araújo e Silva Ferraz, Fernanda Campos de Sousa, Rafaella Resende Andrade, Matteo Barbari

**Affiliations:** 1 Department of Agricultural Engineering, Federal University of Viçosa (UFV), Viçosa 36570-900, MG, Brazil; 2 Department of Engineering, Federal University of Lavras (UFLA), Lavras 37200-900, MG, Brazil; 3 Department of Agricultural Engineering, Federal University of Lavras (UFLA), Lavras 37200-900, MG, Brazil; 4 Department of Agriculture, Food, Environment and Forestry, University of Firenze, 50145 Firenze, Italy

**Keywords:** dairy cattle, confinement systems, thermal comfort, thermal conditioning, geostatistics

## Abstract

**Simple Summary:**

Adequate environmental conditions are essential to ensure the wellbeing and productivity of dairy cattle. The use of compost-bedded pack barn (CBP) systems in dairy farming certainly improves animal welfare conditions, but it is necessary to evaluate and characterize the thermal environment inside the facilities. The main objective of this study was to map the thermal environment inside a CBP system with positive-pressure ventilation. Through mapping, it was possible to identify regions with more challenging conditions for animals in terms of thermal comfort. The results achieved can be used to direct decision-making processes to create adequate environmental conditions for the hosted animals.

**Abstract:**

The objective of this study was to evaluate and characterize the dependence and the spatial and temporal distribution of variables and indices of the thermal environment in an open compost-bedded pack barn system with positive-pressure ventilation (CBPPV) during the winter period. The study was conducted in a CBPPV system located in the Zona da Mata region, Minas Gerais, Brazil. The indoor environment was divided into a mesh composed of 55 equidistant points, where data on dry-bulb air temperature (t_db_) and relative humidity (RH) were collected. The collected data were divided into four periods—dawn, morning, afternoon, and night—and mean values were obtained. To evaluate the thermal microenvironment, the temperature and humidity index (THI) and the specific enthalpy of air (*h*) were used. For spatial dependence analysis, geostatistical techniques were applied. Through the results, a strong spatial dependence was verified for all variables evaluated. Through THI and *h* maps, conditions of thermal comfort were found for dairy cattle. The highest values of t_db_, THI, and *h* were recorded in the afternoon period in the northwest region of the facility (t_db_ = 23.2 °C, THI = 69.7, and *h* = 50.9 kJ∙kg of dry air^−1^).

## 1. Introduction

In dairy cattle production, the use of confinement systems is one of the main strategies applied to improve the thermal environment [1,2]. Its main advantage is to enable the control of environmental conditions and, consequently, ensure an adequate environment for animals to express their maximum productive potential [3,4].

In recent decades, there has been a tendency to use housing systems without restraint of animals in individual stalls; this approach, in addition to ensuring adequate conditions of thermal comfort, allows greater freedom of movement and interaction between animals, as well as reducing greenhouse gas emissions [5]. In Brazilian dairy farms, there has been an increase in the adoption of confinement systems known as compost-bedded pack barns (CBPs). This system makes it possible to ensure improvements in the comfort, productivity, health, and longevity of the herd. In addition, it has a lower implantation cost and greater environmental focus, since it uses less water to clean waste, and produces a compost with desirable agronomic characteristics [2,6,7].

The first CBP systems built in Brazil were designed according to the North American model, idealized for temperate climate conditions [8]. However, given the tropical and subtropical climate conditions present in the Brazilian territory, it has been decided to use these facilities with open sides. In this case, natural ventilation was initially explored. However, in places where the natural ventilation rate is low or the temperature and relative humidity conditions are extreme, it is necessary to use mechanical ventilation systems, either associated or not associated with evaporative adiabatic cooling [9,10,11].

The use of mechanical ventilation systems makes it possible to improve thermal conditions inside the facility (i.e., temperature (t_db_), relative humidity (RH), and air speed (v_air_)), directly influencing the wellbeing and performance of dairy cattle [10]. However, the ventilation systems used in Brazilian CBP facilities need to be evaluated and characterized in detail, so that the necessary adjustments can be made in the design of new facilities and/or the management of existing facilities [12].

In any animal production system, studies regarding the evaluation and characterization of the environment are important, because they make it possible to identify any existing failures, to propose solutions, and to provide valuable information for producers [13]. This evaluation can be performed based on climatic elements, such as t_db_ and RH, which enable us to make a generalized diagnosis. However, the use of thermal comfort indices (TCIs) makes it possible to obtain more representative information, since they consider the combined effect of these elements [14,15].

Because it is easy to obtain, requiring only t_db_ and RH, the temperature and humidity index (THI) has been widely used for evaluation of the thermal environment in milk production systems [2]. Another index widely used to evaluate the thermal environment in this type of system is the specific enthalpy of air (*h*, in kJ∙kg of dry air^−1^), which uses thermodynamic air properties (t_db_ and RH) to estimate the amount of energy contained in a water–vapor mixture [16,17]. The use of this index has been highly appreciated in tropical and subtropical climate regions, where elevated temperatures are recorded most of the year and, therefore, there is high energy expenditure by animals to activate heat dissipation mechanisms [2].

It is important that innovative computational tools are used that can assist in decision making and contribute to improving the environment [18]. Among the tools used, geostatistics stands out, enabling the evaluation of dependence and spatial distribution, and interpretation of the results from the data’s natural structure [19,20]. Although previous studies have carried out the mapping of the thermal environment inside CBP systems, few studies have evaluated this environment under Brazilian winter conditions. The use of this tool in animal ambience is quite satisfactory, and has been carried out by several researchers [10,11,20,21,22,23,24]. In view of the above, the objective of this study was to evaluate and characterize the dependence and spatial distribution of the thermal environment variables and indices in an open compost-bedded pack barn system with positive-pressure ventilation (CBPPV) during the Brazilian winter period.

## 2. Materials and Methods

The study was conducted for three consecutive weeks in July 2021, during the winter period in Brazil. In this climatic season, conditions of high relative humidity are usually observed in the study region—a factor that can cause an increase in bed moisture and compromise the viability of the CBP systems.

The research was approved by the Ethics Committee in Animal Use of the Federal University of Viçosa (protocol code 04/2021 with approval date 16 May 2022). All procedures were performed in accordance with the guidelines recommended by this committee.

### 2.1. Characterization of the Facility and Management Techniques

The experimental data were collected in a facility for dairy cattle confinement, in a compost-bedded pack barn system with positive-pressure ventilation (CBPPV). The facility where the study was carried out is located on a commercial property in the Zona da Mata region, Minas Gerais, Brazil (coordinates 20°46′41″ S and 42°48′51″ W; altitude 670.0 m). The climate is classified as Cwa—subtropical mesothermal, with rainy warm summers, and dry cold winters—according to the Köppen classification [25].

**Figure 1:**
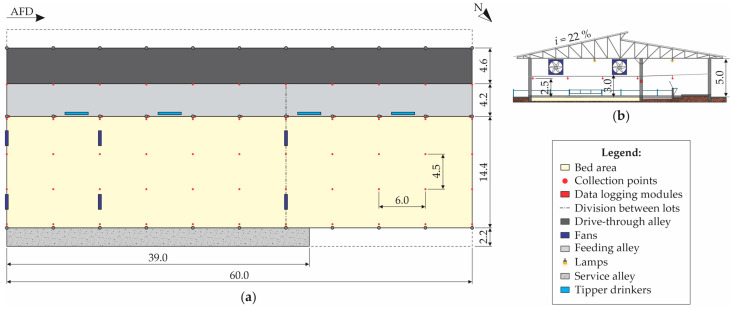
Schematic representation, in (**a**) low plan and (**b**) cross-sectional views, of the compost-bedded pack barn system, with data collection points indicated. AFD—air-flow direction; N—north indication; *i*—roof pitch; dimensions in meters (m).

The facility was built in July 2019, with a southeast–northwest orientation, and the following constructed characteristics: 60.0 m length × 27.6 m width, 5.0 m of right-foot, gable roof with structure and metal roof tiles, central opening with 1.0 m overlap, and 0.8 m eaves. The internal spatial distribution of the CBPVP (Figure 1) is as follows: 864.0 m^2^ bed area (on compacted soil); 252.0 m^2^ feeding alley with a concrete floor, with four tipper drinkers (separated from the pack area by a 1.2 m high concrete wall); 220.0 m^2^ drive-through alley with a concrete floor (containing a single 60.0 m long trough), which is the region where the tractor circulates for food distribution; and 85.8 m^2^ of service alley with a concrete floor.

Ventilation in the CBPPV system was performed by positive pressure, provided by means of six mechanical fans with low volume and high rotation: two three-propeller fans, with 1.52 m diameter, 1.5 hp, and 86,000 m^3^·h^−1^ air flow, installed on the southeast side; and four six-propeller fans, with 1.53 m diameter, 2.0 hp, and 55,000 m^3^·h^−1^ air flow, installed two-by-two along the length of the facility, at 12.0 and 36.0 m, respectively, in relation to the southeast face (Figure 1). The fans were installed 3.0 m high, with a 45° inclination, and remained on continuously (24 h·day^−1^).

The lighting inside the facility was provided by 18 100 W LED lamps, installed 4.8 m above the bed, and distributed throughout the facility: 9 in the center of the bed area and 9 in the border region between the feeding alley and the drive-through alley. The lighting system remained activated only at night (06:00 p.m. to 06:00 a.m.).

The bed area of the system was separated from the feeding alley by a small 0.2 m high wall, with the function of avoiding the passage of bed material to the feeding alley and/or waste from the feeding alley to the bed area. This small wall was present in the five access passages from the bed area to the feeding alley. In the places where tipper drinkers were installed, taller walls (1.2 m) were built to contain the animals, preventing them from having access to water directly from the bed area, which could wet it.

The CBPPV had a bed composed of a mixture of wood shavings and sawdust, with a thickness of approximately 0.6 m. For the bed composition, initially, a 0.3 m thick dry sawdust layer was added which, together with the feces and urine of the animals, started the semi-composting process. The addition of dry substrate was performed whenever the bed moisture was exceedingly high—a condition observed when there was an increase in the animals’ dirtiness, excessive compaction, and consequent anaerobiosis in the compost. Such an addition occurred twice during the experimental period. The present study started about four months after the bed change and, during the experimental period, only minor additions were performed.

The turning of the bed was performed using a hybrid implement (bed rototiller with cultivator, 2.0 m of actuation width, five rods, 0.50 m maximum depth, 540 rpm of maximum rotation, and effective depth of 0.30 m) driven by a tractor (light line, 78 hp, and 2400 rpm of nominal rotation). This operation was performed twice daily (09:00 a.m. and 04:00 p.m.), following the routine established on the farm.

In the normal farm routine, the animals housed in the CBPPV remained distributed in two lots, according to their milk yield. The cows with higher productivity were housed in a specific facility region (Lot 1) with a 518.4 m^2^ bed area (36.0 × 14.4 m), located near the southeast face of the facility. The animals with lower productivity remained housed in the specific facility region bounded by Lot 2, with a 345.6 m^2^ bed area (24.0 × 14.4 m), located near the northwest face of the facility. During the experimental period, 80 lactating Holstein cows (pure of origin (PO), 600 kg average weight) remained housed inside the facility, of which 45 constituted Lot 1 (11.52 m^2^·animal^−1^), and 35 were confined in Lot 2 (9.87 m^2^·animal^−1^).

Throughout the experimental period, the standard routine of activities in the CBPPV system was maintained, with milking and feeding twice a day. The milkings started at 04:00 a.m. and 04:00 p.m., with a 2 h 30 min average duration, and were performed in a 2 × 6 fishbone-type room attached to the CBPPV system. Throughout the daytime, the animals had access to the feeding alley, where food and water were available without restrictions. The feeding alley floor was washed once a day (in the morning), using a flushing system.

### 2.2. Microclimatic Data Acquisition System

The microclimatic data acquisition (dry-bulb air temperature (t_db_) and relative humidity of the air (RH)), both inside the system and in the external environment (meteorological shelter), was performed every 5 min, 24 h·day^−1^, throughout the experimental period, comprising three consecutive weeks (July 2021).

To collect the data in the system, the animal-occupied zone (AOZ), composed of the bed and feeding alley areas, was divided by a regular mesh (6.0 × 4.5 m) composed of 55 equidistant points, with markings made according to the facility’s constructed characteristics (Figure 1a). The sensors were installed 2.5 m above the bed level and the feeding alley floor (Figure 1b), so as to allow the passage of the tractor used for daily turning of the bed.

Measurements of the variables t_db_ and RH were performed using electronic components (DHT22 sensors, model AM2302; temperature measurement range from −40.0 to 80.0 °C, with 0.5 °C accuracy; humidity measurement range from 0 to 100%, with 2% accuracy; Aosong Electronics Co. Ltd., Guangzhou, China) distributed at the 55 collection points throughout the AOZ. For processing and recording the collected data, the mesh was divided into 11 collection lines (CLs), composed of 5 sensors and a data collection and recording module. Each of the 11 data collection and recording modules consisted of an Arduino Uno R3 (ATmega328 microcontroller; 5.0 V supply voltage; 16 MHz clock speed; Atmel Corporation, San Jose, CA, USA) connected to a Data Logger Shield with RTC and SD Reader (SD card slot, integrated real-time clock DS1307; FAT16 or FAT32 card formatting; 3.3 V supply voltage; Dallas Semiconductor, Dallas, TX, USA) and a 16 × 2 LCD Display (I2C Backlight Blue, 5.0 V supply voltage; 4 or 8 bits communication; Beijing Qingyuan Innovation and Technology Development Co. Ltd., Shenzhen, China), according to the methodology adapted by Freitas et al. [26]. The Data Logger Shield with RTC and SD Reader was used to control time and record data on an SD card, while the LCD display was used to visualize the date, time, t_db_, and RH data recorded.

To characterize the external environment near the CBPPV, a meteorological shelter was installed, where t_db_ and RH data were collected. The t_db_ and RH records were taken using a sensor recorder (HOBO^®^, model U14-002; temperature measurement range between −20.00 and 50.00 °C, with 0.21 °C accuracy; relative humidity measurement range between 0 and 100%, with 2.5% accuracy). As well as inside the facility, external data were collected every 5 min, 24 h·day^−1^, throughout the three weeks of the experimental period.

The average daily t_db_ and RH data obtained in four periods of the day were used: dawn (12:00 a.m. to 05:59 a.m.), morning (06:00 a.m. to 11:59 a.m.), afternoon (12:00 p.m. to 05:59 p.m.), and night (06:00 p.m. to 11:59 p.m.). This division was carried out with the objective of identifying the occurrence of critical environmental conditions, according to the methodology adapted by Andrade et al. [27].

### 2.3. Thermal Comfort Evaluation

The initial evaluation of the thermal environment was performed using the climatic elements t_db_ and RH. To this aim, the t_db_ and RH ranges were delimited as optimal, thermoneutral, or critical for lactating Holstein cows. These ranges were established based on studies and technical materials published by various experts [1,15,28,29,30,31,32,33,34]. Table 1 lists the t_db_ and RH ranges established in the present study, as well as the temperature and humidity index (THI) and specific enthalpy of air (*h*) intervals calculated from the t_db_ and RH values considered to be optimal, thermoneutral, and critical for lactating Holstein cows.

The t_db_ and RH data recorded during the winter experimental period were used to calculate the THI and the *h*, applied to the thermal comfort evaluation of dairy cattle housed in the facility.

The THI was calculated using Equation (1), according to the model proposed by Mader et al. [35]:

(1) $$THI=0.8\times t_{db}+RH\times \left(\frac{t_{db}-14.3}{100.0}\right)+46.3$$

The *h* (in kJ·kg of dry air^−1^) was calculated according to the model proposed by Rodrigues et al. [16]:

(2) $$h=1.006\times t_{db}+\frac{RH}{Pa}\times 10^{\left(\frac{7.5\times t_{db}}{237.3+t_{db}}\right)}\times \left(71.28+-0.052\times t_{db}\right)\;$$

where *Pa* is the local barometric pressure (706 mmHg).

### 2.4. Statistical Analyses

#### 2.4.1. Descriptive Analysis of Environmental Data

The initial analysis of the thermal environmental data recorded in the experimental period was performed using descriptive statistics. Primarily, the mean, absolute minimum, and absolute maximum data of t_db_ and RH were obtained, and were used to evaluate the behavior of these variables throughout the winter experimental period. Together, mean hourly values of the variables (t_db_ and RH) and indices (THI and *h*) were calculated, and were applied to the evaluation of the variation in these attributes throughout the day.

Finally, for the average daily data of t_db_, RH, THI, and *h* per period (dawn, morning, afternoon, and night), the mean, median, minimum, maximum, standard deviation (SD), coefficient of variation (CV), kurtosis, and skewness values were obtained. To evaluate the experimental data’s dispersion, the CV classification proposed by Warrick and Nielsen [36] was adopted: CV < 0.12 = low dispersion; 0.12 ≤ CV < 0.24 = moderate dispersion; CV ≥ 0.24 = high dispersion.

#### 2.4.2. Analysis of Variability and Spatial Distribution 

To evaluate the spatial behavior of the variables (t_db_ and RH) and indices (THI and *h*) inside the facility, as well to verify whether they showed spatial dependence, geostatistical techniques were used. Geostatistical analyses were performed using the R Development Core Team computer system [37], through the geoR library [38].

To evaluate the spatial dependence of the variables in the facility’s internal area, semivariogram adjustments were made using the Matheron estimator [39], according to Equation (3):

(3) $$\hat{\gamma }\left(h\right)=\frac{1}{2N\left(h\right)}\overset{N\left(h\right)}{\underset{i=1}{\sum }}{\left[Z\left(X_{i}\right)-Z\left(X_{i}+h\right)\right]}^{2}$$

where $\hat{\gamma }\left(h\right)$ is the semivariance, N(h) is the number of pairs of experimental observations $Z\left(X_{i}\right)$ and $Z\left(X_{i}+h\right)$, and h is the distance between the experimental observations.

The experimental semivariogram adjustments were performed using the methods of ordinary least squares (OLS) and restricted maximum likelihood (REML). For each method, spherical, exponential, and Gaussian models (Equations (4)–(6), respectively) were tested, as described by Vieira et al. [40].

(4) $$\hat{\gamma }\left(h\right)=C_{0}+C_{1}\times \left[\;1.5\times \left(\frac{h}{a}\right)-0.5\times {\left(\frac{h}{a}\right)}^{3}\right],\;\mathrm{if}\;h\;\leq a\;\mathrm{ou}\;\hat{\gamma }\left(h\right)=C_{0}+C_{1},\;\mathrm{se}\;h>a$$

(5) $$\hat{\gamma }\left(h\right)=C_{0}+C_{1}\times \left[\;1-e^{\left(\frac{-3h}{a}\right)}\right]$$

(6) $$\hat{\gamma }\left(h\right)=C_{0}+C_{1}\times \left\{\;1-e^{\left[-3\times {\left(\frac{h}{a}\right)}^{2}\right]}\right\}$$

where $C_{0}$ is the nugget effect, $C_{1}$ is the contribution, and a is the range.

For the evaluation and choice of the adjustments obtained, cross-validation procedures were performed, and the mean error (ME), mean-error standard deviation (SD_M_), reduced error (RE), and reduced-error standard deviation (SD_R_) were calculated. From the adjustments obtained using the methods and models described, for each variable, the adjustment was chosen in which the ME and RE were closer to zero, while the SD_M_ and SD_R_ were closer to one, as recommended by Isaaks and Srivastava [41].

From the mathematical models $\hat{\gamma }\left(h\right)$ chosen for each variable, the following coefficients of the semivariogram theoretical model were obtained: nugget effect ($C_{0}$), contribution ($C_{1}$), sill ($C_{0}+C_{1}$), range (a), and practical range (*a*′).

The verification of the occurrence of spatial dependence was performed using the spatial dependence index (SDI), determined by the ratio between $C_{0}$ and $C_{0}+C_{1}$. For SDI analysis, the classification of Cambardella et al. [42], which considers semivariograms with SDI ≤ 0.25 as showing strong spatial dependence, semivariograms with 0.25 < SDI ≤ 0.75 as showing moderate spatial dependence, and semivariograms with SDI > 0.75 as showing weak spatial dependence.

Finally, after the theoretical semivariograms with better adjustments were chosen, as well as the occurrence of spatial dependence, the ordinary kriging technique was used to predict the levels of variables and indices in places that were not sampled inside the facility. From the interpolated data, surface response maps were generated using the computational program ArcGIS^®^, version 10.1, with license for use by the Department of Agricultural Engineering of the Federal University of Viçosa.

## 3. Results and Discussion

**Figure 2:**
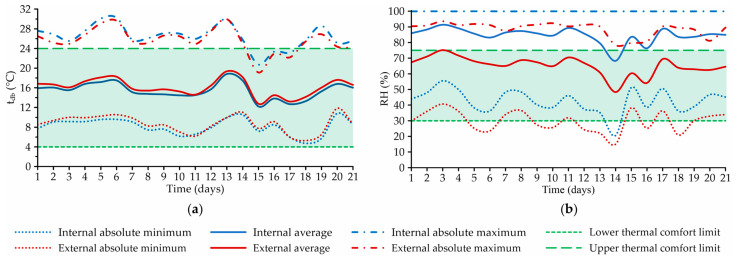
Daily curves of the mean, absolute minimum, and absolute maximum values of (**a**) dry-bulb air temperature (t_db_, in °C) and (**b**) relative air humidity (RH, in %) in the internal and external environments of the open compost-bedded pack barn system throughout the experimental period.

Figure 2 illustrates the daily curves of the mean, absolute minimum, and absolute maximum values of t_db_ (in °C) and RH (in %), recorded inside and outside the facility.

Regarding t_db_, it can be observed that the internal and external data curves (mean, absolute minimum, and absolute maximum values) had similar profiles throughout the experimental period, being close to one another (Figure 2a). This was already expected, since it was an open facility, in which there is usually a high correlation between internal and external temperatures [43].

The absolute daily minimum t_db_ values recorded over the winter experimental period were always higher than 4.0 °C (indoor and outdoor), and the lowest were recorded inside the facility. The lowest values were recorded on the 18th day of collection, with values of 4.7 °C in the internal environment and 5.3 °C in the external environment.

Observing the mean and absolute maximum values of t_db_ recorded inside and outside the facility (Figure 2a), it can be verified that the curves had behaviors similar to what was observed for the absolute minimum t_db_. This means that on the days when the lowest absolute minimum t_db_ values were recorded, lower levels of mean t_db_ and absolute maximum t_db_ were also observed. In fact, on days with colder dawns, there was also a tendency to record lower mean and maximum temperatures.

The lowest mean values of t_db_ were recorded on the 15th day of collection, with values of 12.2 °C in the internal environment and 12.8 °C in the external environment, while the highest values were observed on the 13th day of collection (18.8 °C in the internal environment, and 19.4 °C in the external environment). For the absolute maximum t_db_, the most critical condition occurred on the 6th day of collection, when the highest internal and external values (30.3 and 29.6 °C, respectively) were recorded.

Regarding the minimum t_db_ levels, it was possible to observe that the values recorded inside the system were higher than the lower thermal comfort limit (LTCL) for lactating dairy cattle (4.0 °C; see Table 1). Thus, it can be concluded that in the winter trial the lodged animals were not exposed to hypothermic conditions, where they would not be able to produce enough heat to keep their body temperature within the ideal range [15,44].

On the other hand, on most days of the experimental period, the absolute maximum t_db_ values were recorded above the upper thermal comfort limit (UTCL) established for lactating dairy cattle (24.0 °C; see Table 1). This means that at some point the t_db_ was above the maximum critical temperature considered for the housed animals. When this happens, the temperature control mechanisms of the animals are not able to ensure sufficient cooling to maintain body temperature within the ideal range, leading to thermal stress, which can generate productive losses and reduce the milk quality [15,45]. The observed results indicate that, even during the winter period, the animals may have been exposed to thermal stress conditions by hyperthermia at some points. Thus, it is recommended that interventions should be performed - such as wetting of animals in the feeding alley, and the use of low-static-pressure ventilation systems - to avoid productive losses, as suggested by Mondaca et al. [46].

Regarding RH (Figure 2b), it is possible to verify that the behaviors of the mean and absolute minimum data curves were similar, whereas the absolute maximum RH profile inside the system was constant throughout the period (RH ≅ 100.0%), and had slight variation outside the facility. Like t_db_, RH levels had variable behavior over time, with absolute minimum and maximum values equal to 20.6 and 100.0%, respectively. The values recorded inside the system were always higher than those observed in the external environment (meteorological shelter), with an average difference of 14.5%. It can be inferred that the occurrence of higher levels of RH in the indoor environment was due to moisture released during the process of bed semi-composting, evaporation of water from tipper drinkers, and release of water by respiration and animal waste [10,21].

During the experimental period, only on the 14th day of collection was the recorded RH value below the LTCL for lactating dairy cattle (30.0%) [32]. On this day, the absolute minimum RH values recorded in the internal and external environments were 20.6 and 15.0%, respectively. Despite favoring heat dissipation, the low RH levels recorded may have caused dryness of the mucous and airways of the housed animals [47].

The lowest mean RH levels were recorded on the 14th collection day (68.3% in the internal environment, and 48.4% in the external environment), while the highest were observed on the 3rd collection day (91.4% in the internal environment, and 75.2% in the external environment). On most days of the experimental period, the mean RH within the system was above the UTCL recommended by the literature for lactating dairy cattle (75.0%) [29]. Only on the 14th day of collection was there a mean RH value lower than the UTCL (68.3%). In addition to hindering heat dissipation, high RH levels can cause several problems for producers, such as increased respiratory disease rates (pneumonia, bronchitis, etc.), bed moisture, dirt rates, mastitis, somatic cell counts, etc. [2,8,15,48]. The recording of high RH values is an indication that it is necessary to carry out interventions in this system, with the objective of improving the environment for the hosted animals.

**Figure 3:**
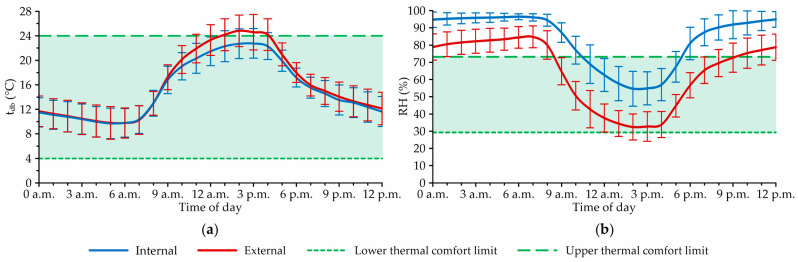
Average hourly profiles, with standard deviations, of (**a**) dry-bulb air temperature (t_db_, in °C) and (**b**) relative humidity (RH, in %) throughout the day in the internal and external environments of the facility, during the experimental period.

The average hourly t_db_ and RH curves in the internal and external environments of the system are illustrated in Figure 3.

Through Figure 3a, it can be observed that the average hourly t_db_ values inside and outside the system presented similar profiles, but the external t_db_ values were higher than the internal ones most of the time. It is also possible to note that between 12:00 a.m. and 09:00 a.m., the average t_db_ values inside the system were close to those recorded outside the facility and, from this time onwards (09:00 a.m. to 09:00 p.m.), the temperature levels inside the facility tended to be lower (average difference of 1.2 °C between the internal and external environments).

The t_db_ values recorded within the facility in this study differ from those portrayed by Pilatti et al. [49], who observed the occurrence of higher t_db_ values within CBP systems when compared to the external environment. In this case, it can be inferred that the use of mechanical ventilation (LVHS) made it possible to reduce the t_db_ levels inside the system at the hottest times of day, as portrayed by Oliveira et al. [21] in a study conducted in Minas Gerais, Brazil.

Regarding RH, the average hourly values inside the system were usually higher than those recorded outside the facility (Figure 3b). It can be observed that the average hourly RH curves showed constant differences throughout the day, with internal levels about 23.6% higher than the external ones. In open CBP systems, RH levels are influenced by conditions inside (bed conditions, ventilation rate, hosted animal density, etc.) and outside (local weather conditions, time of year, etc.) [2]. Therefore, it can be concluded that the higher RH values recorded throughout the day inside the facility are an indication that the internal environment had a great contribution to the elevation of this attribute.

**Figure 4:**
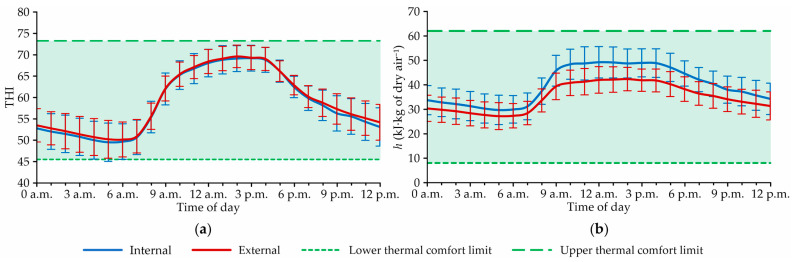
Average hourly profiles of the (**a**) temperature and humidity index (THI) and (**b**) specific enthalpy of air (*h*, in kJ∙kg of dry air^−1^) throughout the day in the internal and external facility environments, during the experimental period.

Figure 4 illustrates average hourly curves of the temperature and humidity index (THI) and specific enthalpy of air (*h*, in kJ∙kg of dry air^−1^), obtained during the winter period, in the internal and external environments of the CBPPV system.

Through Figure 4a, it can be observed that the average hourly THI curves had profiles similar to those observed for t_db_, which was expected, since this attribute has greater weight for THI composition. For these curves, it was found that the internal and external values that we obtained remained practically the same throughout the day. In this case, the occurrence of hours with higher external THI values was not observed, due to the high internal RH values, which returned internal THI values close to those observed outside the facility, even at times when higher external t_db_ was recorded.

Throughout the day (Figure 4a), it can be observed that the calculated THI values remained within the range established in this study as thermal comfort for lactating dairy cattle (46.0 ≤ THI < 74.0; see Table 1), and always below 74.0—the upper THI limit recommended by Mader et al. [35]. Therefore, using this index, it can be concluded that during the experimental winter period, the housed animals were not exposed to thermal stress conditions within the CBPPV system.

Regarding *h* (Figure 4b), it was found that the average hourly curves of the internal and external values had similar profiles, but that the internal levels were always higher than those obtained outside the system. The occurrence of higher *h* levels inside the facility was due to the high RH values recorded in this environment, which consequently increased the *h* values obtained.

Even if higher *h* levels were obtained within the system, the observed values remained within the thermoneutral range for lactating dairy cattle (8.0 ≤ *h* < 62.0 kJ∙kg of dry air^−1^; see Table 1). Therefore, it can be concluded that the amount of heat present inside the facility during this period did not represent a problem in terms of thermal comfort.

Table 2 lists data from the descriptive analysis of the variables (t_db_ and RH) and indices (THI and h) recorded within the CBPPV system in the periods of dawn (12:00 a.m. to 05:59 a.m.), morning (06:00 a.m. to 11:59 a.m.), afternoon (12:00 a.m. to 05:59 p.m.), and night (06:00 p.m. to 11:59 p.m.).

During the experimental period, the minimum and maximum t_db_ values were recorded in the morning and afternoon periods, respectively (10.5 ± 0.2 and 21.7 ± 0.3 °C, respectively) (Table 2). As expected, it was observed that the RH data had inverse behavior to the t_db_ data, with higher values recorded in the dawn period (98.1 ± 0.7%) and lower values in the afternoon period (61.7 ± 1.8%).

As THI and *h* are obtained from the combined effect of t_db_ and RH, it was observed that the variations in values obtained for these indices were similar to those of t_db_, which had greater weight in both compositions. Thus, the highest means occurred in the afternoon period (THI = 68.3 ± 0.4 and *h* = 48.7 ± 0.8 kJ∙kg of dry air^−1^), while the lowest were recorded in the dawn period (THI = 50.9 ± 0.3 and *h* = 31.2 ± 0.4 kJ∙kg of dry air^−1^).

For all variables and in all periods evaluated, it can be observed that the mean and median values were close to one another, indicating that the data did not present marked asymmetry. Therefore, it can be assumed that the distributions of the average hourly recorded data were approximately normal [50].

Evaluating the data dispersion listed in Table 2 based on the coefficient of variation (CV) classification proposed by Warrick and Nielsen [36], it was found that for all average hourly data (t_db_, RH, THI and h) the CV values were lower than 0.12, indicating low dispersion. The low CV values obtained are also an indication that the variables had approximately uniform distribution. These results corroborate those described by Andrade et al. [27], who evaluated a closed CBP system in the same region where this study was conducted, and obtained CV values below 0.12.

Considering the thermoneutral range for lactating dairy cattle (4.0 ≤ t_db_ < 24.0 °C; see Table 1), it can be observed that in all of the periods evaluated, the average hourly levels of t_db_ were within the range considered appropriate for the species (Table 2). Even in the hottest period of the day, the t_db_ values recorded inside the facility were lower than the UTCL and, therefore, did not indicate thermal discomfort.

Except for the afternoon period (Table 2), it was observed that mean hourly RH levels obtained were above the superior threshold of thermal comfort recommended for lactating dairy cattle (30.0 ≤ RH < 75.0%; see Table 1). However, it should be noted that the occurrence of high RH values throughout the winter trial period did not impair the productive performance of the animals, since the mean t_db_ levels remained below the temperature considered critical for lactating dairy cattle (24.0 °C; see Table 1).

Regarding THI, Table 2 shows that the mean values in all periods evaluated were within the thermal comfort range established in this study for lactating dairy cattle (46.0 ≤ THI < 74.0; see Table 1). Even in the afternoon period, in which the highest t_db_ values were recorded, the combination of these with low RH values caused THI values below 74.0, indicating thermal comfort. For *h*, mean hourly values were also obtained within the thermal comfort range for lactating dairy cattle (8.0 ≤ *h* < 62.0 kJ∙kg of dry air^−1^; see Table 1). From a global analysis of variables (t_db_, RH, THI, and *h*), it can be seen that the thermal environment within the studied CBP was within the ranges considered adequate for cows to produce milk; for this reason, the energy expenditure for the activation of heat dissipation mechanisms was minimal throughout the entire winter experimental period.

Table 3 lists the methods, models, and parameters estimated from the experimental semivariograms adjusted for the variables and indices of the thermal environment evaluated during the winter experimental period.

The best experimental semivariogram adjustments were obtained using the spherical and exponential models which, according to Webster and Oliver [51], are the most frequently used in geostatistics. Based on cross-validation data (Table 3), it was found that the adjustments obtained were adequate, given that ME and RE values close to zero were obtained (<0.0100), as well as SD_R_ values close to one, as recommended by Isaaks and Srivastava [41].

Among the geostatistics parameters, one of the main parameters is the nugget effect (*C*_0_), which refers to unexplained variability considering the distance between sampled points [52]. For the variables and indices evaluated (Table 3), the *C*_0_ values were mostly low (close or equal to zero), indicating that the variables and indices evaluated present low unexplained variability, and that the adjusted semivariograms do not have discontinuity.

On the other hand, as the discontinuity represented by *C*_0_ can be attributed to several factors (e.g., errors of collections and/or analyses, local variations etc.), and it is not possible to quantify the contribution of each factor, it is important that other forms of evaluation of *C*_0_ are used [53]. One of these ways is to use the spatial dependence index (SDI) and express *C*_0_ in relation to the sill (*C*_0_ + *C*_1_), making it possible to make correlations through the classification of Cambardella et al. [42].

When the SDI was used to evaluate the contribution of unexplained variability in the composition of the level (Table 3), it was found that the SDI values obtained were lower than 0.2500, indicating the occurrence of strong spatial dependence. The highest SDI values were observed for the variables t_db_ (dawn) and RH (morning), in which values equal to 0.1229 and 0.1305 were obtained, respectively. Therefore, it can be concluded that the results obtained with the use of ordinary kriging are representative of the variables, since the low contributions of the nugget effect to the sill return better results with the interpolation techniques used by ordinary kriging [54].

Another very important parameter is the range (*a*), which represents the influence distance of an observation by differentiating correlated samples (structured) from independent samples (random) [55]. In all cases evaluated in this study, the *a* values obtained were greater than the shortest distance between sampled points (4.5 m), and the occurrence of spatial dependence was observed (Table 3). The lowest a values were observed for RH (afternoon, *a* = 5.0397 m), THI (morning, *a* = 5.0215 m), and h (morning, *a* = 5.0008 m). By analyzing the *a* values, it becomes evident that the distance between sampling points was adequate.

After verifying the occurrence of strong spatial dependence for all variables (Table 3), it was possible to use ordinary kriging to obtain the data from unsampled points. From the kriging data, spatial distribution maps were generated, which made it possible to identify regions with higher and lower levels of the studied variables [56].

**Figure 5:**
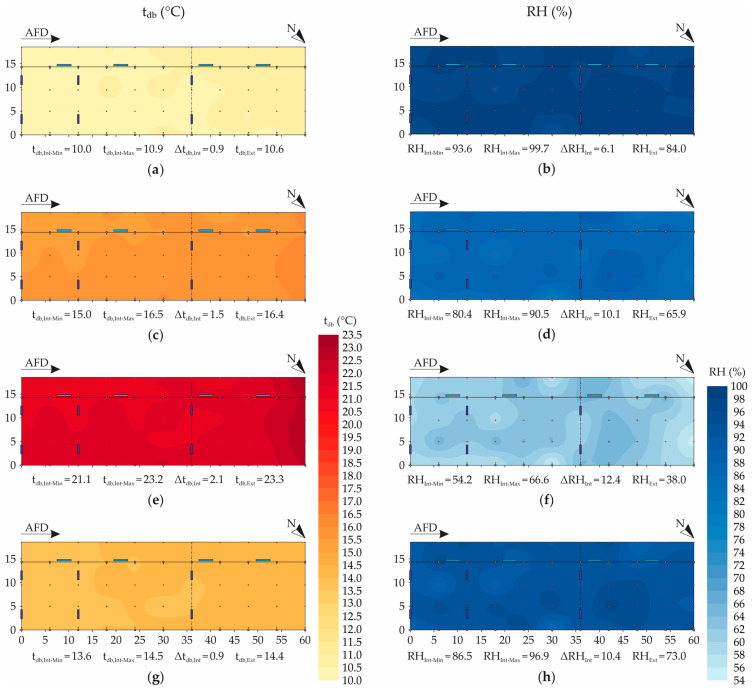
Spatial distribution of dry-bulb air temperature (t_db_, in °C) and relative humidity of air (RH, in %): (**a**,**b**) dawn (12:00 a.m. to 05:59 a.m.), (**c**,**d**) morning (06:00 a.m. to 11:59 a.m.), (**e**,**f**) afternoon (12:00 a.m. to 05:59 p.m.), (**g**,**h**) and night (06:00 p.m. to 11:59 p.m.). AFD—air-flow direction; N—north indication; t_db,Int-Min_—minimum internal dry-bulb air temperature; t_db,Int-Max_—maximum internal dry-bulb air temperature; Δt_db,Int_—variation of internal dry-bulb air temperature; t_db-Ext_—mean external dry-bulb air temperature; RH_Int-Min_—minimum internal relative humidity of air; RH_Int-Max_—maximum internal relative humidity of air; ΔRH_Int_—variation of internal relative humidity of air; RH_Ext_—mean internal relative humidity of air; dimensions in meters (m).

Figure 5 illustrates the spatial distribution maps of the variables dry-bulb air temperature (t_db_) and relative humidity of air (RH) during the winter experimental period, in the dawn, morning, afternoon, and night.

Figure 5a,c,e,g show that there was a low spatial variability of the mean t_db_ values, and temperature values close to those recorded outside the facility were observed (meteorological shelter). In the dawn, morning, and night periods, low variation amplitudes were obtained (0.9, 1.4, and 0.9 °C, respectively). In the afternoon period, the t_db_ amplitude was slightly higher (2.1 °C), with average values between 21.1 and 23.2 °C.

Even though low variation amplitudes were observed, the formation of a t_db_ gradient throughout the facility was observed, with lower and higher t_db_ values always occurring in regions near the southeast and northwest faces, respectively (Figure 5a,c,e). The occurrence of a region with higher average temperatures is an indication that the ventilation system used was not effective in ensuring homogeneous thermal conditions, even during the coldest season (winter).

For low-volume and high-speed (LVHS) mechanical fans, such as those used in the CBPPV system, the distance between the longitudinal ventilation lines should be between 12.0 and 18.0 m, and it is recommended that it be 12.0 m [2]. Near the southeast face of the studied facility, four ventilators (0.0 and 12.0 m shares, from southeast to northwest) remained connected 24 h·day^−1^ and, in turn, made it possible to reduce the t_db_ in their operational areas [21,57]. However, in the rest of the facility area, only two more fans were present (dimension 36.0 m, from southeast to northwest), so the distance between the second and third lines and the northwest end (24.0 m) was greater than recommended. Thus, it is inferable that the number and arrangement of fans used were not satisfactory to reduce the mean t_db_ values.

Specifically in relation to the region near the northwest face of the facility, the most notable situation occurred in the afternoon, in which direct incident solar radiation was observed within the facility and, consequently, higher mean t_db_ values were recorded (23.2 °C; see Figure 5e). The direct incident solar radiation in this region occurred due to two factors: facility orientation, and the absence of closure of the upper part of the northwest face. The facility evaluated was oriented in the southeast–northwest direction and, for this reason, some regions of the building were prone to receiving direct incident solar radiation at some times of the day during the winter [2], as verified through the t_db_ spatial distribution maps (Figure 5e). In these cases, it is recommended that closing devices are installed on the top, near the roof, to minimize the direct incident solar radiation inside the facility [58]. On the other hand, as this was only a small fraction of the area, it is recommended that secondary modifications be applied, such as installation of closing structures or shade nets in the upper part, which block the entrance of solar radiation and, therefore, make it possible to reduce the average t_db_ levels in this region [15].

Considering the thermoneutral temperature range for lactating dairy cattle that was established in this study (4.0 ≤ t_db_ < 24.0 °C; see Table 1), it can be observed that the average hourly t_db_ values recorded during the winter period were within the range considered ideal (Figure 5a,c,e,g). The highest mean t_db_ levels were recorded in the afternoon period (23.2 °C) (Figure 5e), and occurred in a region near the northwest face of the facility which, as already mentioned, received direct incident solar radiation.

Even though at the northwest face of the facility no mean t_db_ values above the UTCL were recorded, this region can be deprecated by the animals in the hottest period of the day (afternoon). When higher t_db_ values are recorded in each location, animals tend to reject the region in question, and group in places with lower t_db_ levels and/or higher air velocities. With this, there is a tendency to increase thermal discomfort, the risk of accidents by tramping of teats and tails, bedding compaction, and quality deterioration [2].

Certainly, t_db_ exerts a strong influence on the thermal comfort and productive performance of dairy cattle, but this variable should always be evaluated in association with RH [15,29]. Through Figure 5b,d,f,h, it can be observed that the RH spatial distribution maps denoted greater spatial variability, in accordance with what was observed in other studies conducted in CBP systems [21,27], with the occurrence of high RH amplitudes. In the dawn, morning, afternoon, and night periods, ranges of 6.2, 10.1, 12.4, and 10.4% were observed, respectively, indicating the heterogeneous distribution of RH inside the facility.

In the dawn period (Figure 5b), it was possible to observe that there were more homogeneous conditions (93.6 < RH ≤ 99.7%), while in the afternoon period (Figure 5f) the RH distribution was more heterogeneous (54.2 < RH ≤ 66.6%). During the daytime periods (morning and afternoon), there was a tendency to have lower RH levels in the peripheral regions of the facility—especially near the northwest face (Figure 5d,f). In these two periods, it can be confirmed that the recording of lower RH values was due to the direct incident solar radiation and the influence of the external environmental conditions, which contributed to the reduction in RH in the internal environment.

In the night period (06:00 p.m. to 11:59 p.m., Figure 5f), an increase in RH was observed due to the influence of the external environment and the bed conditions. In the dawn (12:00 a.m. to 05:59 a.m., Figure 5b), the increasing RH trend was maintained, and a more uniform distribution was observed (ΔRH_Int_ = 6.2%, lowest variation between periods).

Considering the RH range established in this study as thermoneutral for lactating dairy cows (30.0 ≤ RH < 75.0%; see Table 1), it can be observed that the mean RH levels within the evaluated system were higher than the UTCL in most periods (dawn, morning, and night), being below 75.0% only in the afternoon period (Figure 5f). Since the evaluated system did not use adiabatic evaporative cooling systems (AECSs), high RH levels may represent a problem for the semi-composting process and bed management, as well as animal health and milk quality [2,8]. In fact, in this type of system there is a tendency to obtain elevated RH levels in the internal environment, because of steam released during the semi-composting process, evaporation and water spillage from tipper drinkers, and the animals’ respiration and waste [10,27]. For this reason, it is necessary to pay greater attention to bed management, with more revolving operations, along with the addition and incorporation of more dry bedding material, to reduce bed moisture and keep the resting area dry, hygienic, and comfortable for the animals [8].

**Figure 6:**
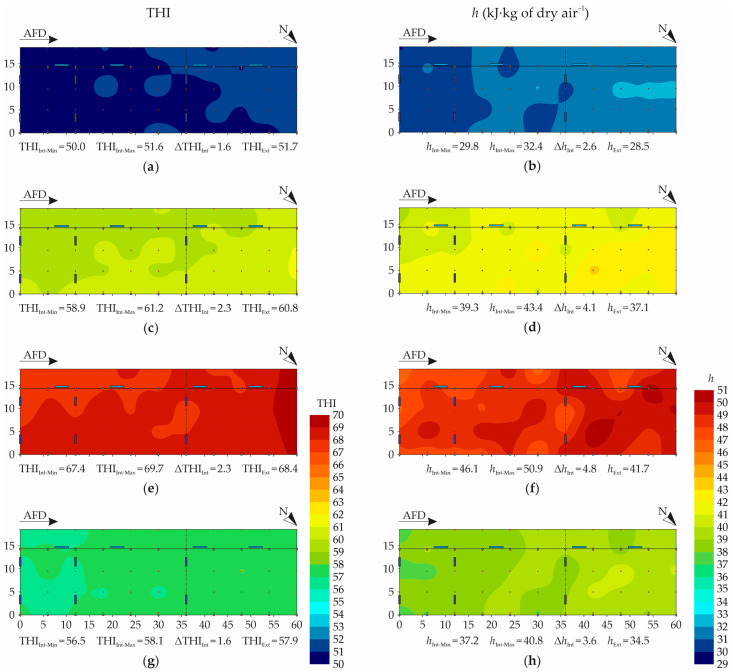
Spatial distribution of temperature and humidity index (THI) and specific enthalpy of air (*h*, in kJ∙kg of dry air^−1^): (**a**,**b**) dawn (12:00 a.m. to 05:59 a.m.), (**c**,**d**) morning (06:00 a.m. to 11:59 a.m.), (**e**,**f**) afternoon (12:00 a.m. to 05:59 p.m.), (**g**,**h**) and night (06:00 p.m. to 11:59 p.m.). AFD—air-flow direction; N—north indication; THI_Int-Min_—minimum internal temperature and humidity index; THI_Int-Max_—maximum internal temperature and humidity index; ΔTHI_Int_—variation of internal temperature and humidity index; THI_Ext_—mean external temperature and humidity index; *h*_Int-Min_—minimum internal specific enthalpy of air; *h*_Int-Max_—maximum internal specific enthalpy of air; Δ*h*_Int_—variation of internal specific enthalpy of air; *h*_Ext_—mean external specific enthalpy of air; dimensions in meters (m).

The THI and *h* spatial distribution maps are illustrated in Figure 6. As can be observed in Figure 6a,c,e,g, the mean THI values’ spatial distributions had behaviors similar to those observed for t_db_, with low spatial variability. The variation amplitudes recorded were low in the four periods evaluated (1.6, 2.3, 2.4, and 1.6, in the dawn, morning, afternoon, and night, respectively).

Similarly to t_db_ (Figure 5), it was observed that in the periods with greater variation amplitude (morning and afternoon), there was the formation of small gradients throughout the facility, with lower values observed near the southeast face and higher values in the northwest region (Figure 6). From Figure 6, it can be inferred that the ventilation system installed on site was not effective in promoting homogeneous thermal comfort conditions. Together, the absence of closing structures in the upper northwest face region of the facility caused direct solar radiation to enter, increasing the t_db_ values and, consequently, the THI.

In all periods evaluated (Figure 6a,c,e,g), the THI values obtained were within the thermoneutral range for lactating dairy cows (46.0 ≤ THI < 74.0; see Table 1), as was expected for the season and climatic region under study. The situation closest to the upper THI limit occurred in the afternoon period, in which the occurrence of a region with THI ≥ 69.0 was observed.

The achievement of THI conditions considered to be thermally comfortable was due to the low t_db_ values recorded inside the facility (Figure 5a,c,e,g), which are common in winter. On the other hand, one cannot forget the elevated RH levels observed in the dawn, morning, and night periods (Figure 5b,d,h) which, although not indicative of thermal stress, may represent a problem for bedding management, herd health, and milk quality.

The THI results achieved in this study (Figure 6a,c,e,g) corroborate those observed by Andrade et al. [27], who evaluated the microclimatic variables in a closed CBP system with a tunnel-mode ventilation associated with AECSs, in the same climatic region as this study. In the latter study, it was observed that the highest mean THI levels occurred in the afternoon period, but were always lower than 74.0, being within the thermal comfort range for lactating dairy cows. Based on the results obtained in the present study, it can be concluded that in this region it is not necessary to make use of AECSs during the winter period, since the conditions observed in open CBP systems already meet the animals’ thermal needs.

Studies characterizing thermal comfort in open CBP systems with mechanical ventilation (LVHS) were also conducted in southern Minas Gerais by Mota et al. [59]. During the winter period, the authors obtained mean THI values of 64.6 and 66.2 in the morning and afternoon periods, respectively (with ventilators on). The results obtained in the present study are also consistent with those reported by Mota et al.

As shown in Figure 6b,d,f,g, it was verified that the *h* distributions were relatively uniform, with the variation amplitudes throughout the facility always being lower than 5.0 kJ∙kg of dry air^−1^. The highest *h* values were obtained in the afternoon (*h*_Int−Max_ = 50.9 kJ∙kg of dry air^−1^), which also had greater variation amplitude (Δ*h*_Int_ = 4.8 kJ∙kg of dry air^−1^). On the other hand, softer distributions were observed in the dawn and night periods, in which lower variation amplitudes were obtained (2.6 and 3.5 kJ∙kg of dry air^−1^, respectively).

In all periods evaluated, it was observed that a small *h* gradient occurred throughout the facility (Figure 5b,d,f,h), with lower levels in the vicinity of the southeast face, and higher levels in the northwest region. The occurrence of higher *h* levels in the vicinity of the northwest face was due to the direct incident solar radiation in this region of the facility which, as already widely discussed, caused the increase in the mean t_db_ values (Figure 5a,c,e,g) and, consequently, in *h* (Figure 5b,d,f,h).

Based on the *h* spatial distribution maps (Figure 5b,d,f,h), it was found that the average hourly *h* levels obtained inside the system during the winter trial period were within the range considered suitable for lactating dairy cows (8.0 ≤ *h* < 62.0 kJ∙kg of dry air^−1^; see Table 1). Therefore, it can be inferred that the levels of heat present inside the facility during this period did not represent a problem in terms of thermal comfort.

The *h* results obtained in this study (Figure 5b,d,f,h) corroborate those described by Andrade et al. [27], who evaluated the thermal conditions during the winter period within a closed CBP system. The authors observed the occurrence of lower *h* values in the dawn period (32.6 ± 0.7 kJ∙kg of dry air^−1^) and higher values in the afternoon period (53.0 ± 0.7 kJ∙kg of dry air^−1^). In the study conducted by the authors, the occurrence of higher *h* values in the afternoon period was due to the high RH levels recorded (76.6 ± 2.4%), which justified using the AECS.

## 4. Conclusions

The temporal analysis of the microenvironment data allowed us to verify that, throughout the study period, the average dry-bulb air temperature (t_db_) remained within the thermal comfort range (4.0 ≤ t_db_ < 24.0 °C), although on most days the absolute maximum t_db_ reached values outside the thermal comfort zone for lactating dairy cows at some times. It was also found that the average relative air humidity (RH) was above the thermal comfort range (30.0 ≤ RH < 75.0%) on most days (from 12:00 a.m. to 10:00 a.m. and from 05:00 p.m. to 12:00 p.m.). However, through the temperature and humidity index (THI) and specific enthalpy of air (*h*), which consider the combination of these two factors, adequate thermal comfort conditions were indicated at all times evaluated.

The application of geostatistical techniques allowed us to verify and characterize the occurrence of spatial dependence of the variables evaluated. For all variables, a strong spatial dependence was observed, making it possible to apply interpolation techniques via ordinary kriging and generate spatial distribution maps.

Through the generated maps, it was possible to observe that the variables (t_db_ and RH) and the indices studied (THI and *h*) presented spatial variability. The t_db_ and RH had variable distribution throughout the facility, concentrated in the morning and afternoon periods, but their combination returned THI and *h* maps with distribution within the thermal comfort range. The highest t_db_, THI, and *h* levels were recorded in the afternoon period in the northwest area (t_db_ = 23.2 °C, THI = 69.7, and *h* = 50.9 kJ∙kg of dry air^−1^), which received direct incident solar radiation, while the lowest values were recorded in the dawn period, in areas close to the fans (t_db_ = 10.0 °C, THI = 50.0 and *h* = 29.8 kJ∙kg of dry air^−1^).

The results found in this study describe the environmental thermal conditions inside a CBP system with positive-pressure ventilation during the winter period in Brazil. It is recommended that new mapping studies be carried out in facilities with different construction typologies, in different regions, and at different times of the year.

## Figures and Tables

**Table 1:** Thermal comfort classification for lactating Holstein cows.

t_db_ (°C)	RH (%)	THI	*h* (kJ·kg of dry air^−1^)	Classification
t_db_ < 4.0	RH < 30.0	THI < 46.0	*h* < 8.0	Critical zone
4.0 ≤ t_db_ < 12.0	30.0 ≤ RH < 50.0	46.0 ≤ THI < 55.0	8.0 ≤ *h* < 24.0	Thermoneutral zone
12.0 ≤ t_db_ < 18.0	50.0 ≤ RH < 60.0	55.0 ≤ THI < 63.0	24.0 ≤ *h* < 39.0	Optimal zone
18.0 ≤ t_db_ < 24.0	60.0 ≤ RH < 75.0	63.0 ≤ THI < 74.0	39 ≤ *h* < 62.0	Thermoneutral zone
t_db_ ≥ 24.0	RH ≥ 75.0	THI ≥ 74.0	*h* ≥ 62.0	Critical zone

t_db_—Dry-bulb air temperature; RH—relative humidity of the air; THI—temperature and humidity index; *h*—specific enthalpy of air.

**Table 2:** Descriptive analysis of the average hourly values of the variables dry-bulb air temperature (t_db_, in °C), relative humidity (RH, in %), temperature and humidity index (THI), and specific enthalpy of air (*h*, in kJ∙kg of dry air^−1^) inside the compost-bedded pack barn system.

Variable	Period	Mean	Median	Minimum	Maximum	Standard Deviation	Coefficient of Variation	Kurtosis	Skewness
t_db_	Dawn	10.5	10.5	10.0	10.9	0.2	0.02	2.28	0.04
	Morning	15.7	15.6	15.0	16.4	0.2	0.01	3.60	0.43
	Afternoon	21.7	21.6	21.1	23.2	0.3	0.01	6.19	1.78
	Night	14.1	14.0	13.6	14.5	0.2	0.01	2.13	0.03
RH	Dawn	98.1	98.2	93.6	99.7	0.7	0.01	6.85	−1.38
	Morning	86.7	86.9	80.4	90.5	1.2	0.01	3.91	−0.46
	Afternoon	61.7	61.8	54.2	66.6	1.8	0.03	3.18	−0.48
	Night	92.9	92.9	86.5	96.9	1.3	0.01	4.28	−0.43
THI	Dawn	50.9	50.9	50.0	51.6	0.3	0.01	2.27	0.05
	Morning	60.0	60.0	58.9	61.2	0.3	0.01	3.41	0.34
	Afternoon	68.3	68.2	67.3	69.7	0.4	0.01	4.42	1.17
	Night	57.3	57.3	56.5	58.1	0.3	0.01	2.15	0.03
*h*	Dawn	31.2	31.2	29.8	32.4	0.4	0.01	2.23	0.14
	Morning	41.5	41.5	39.3	43.4	0.6	0.01	2.86	−0.03
	Afternoon	48.7	48.7	46.1	50.9	0.8	0.02	2.44	−0.02
	Night	39.0	39.0	37.2	40.7	0.6	0.01	2.61	0.00

**Table 3:** Methods, models, and parameters estimated from the semivariograms adjusted for dry-bulb air temperature (t_db_), relative humidity (RH), temperature and humidity index (THI), and specific enthalpy of air (*h*).

Variable	Period	Method	Model	*C*_0_	*C*_1_	*C*_0_ + *C*_1_	*a*	*a’*	SDI	ME	SD_M_	RE	SD_R_
t_db_	Dawn	OLS	Spherical	0.0052	0.0371	0.0423	6.6380	6.6380	0.1229	−0.0001	0.2274	−0.0002	1.1110
	Morning	OLS	Spherical	0.0097	0.0819	0.0916	5.8736	5.8736	0.1059	0.0000	0.2927	0.0001	0.9628
	Afternoon	REML	Spherical	0.0180	0.2166	0.2346	40.6683	40.6683	0.0767	0.0048	0.2517	0.0094	1.0209
	Night	OLS	Spherical	0.0056	0.0459	0.0515	5.0819	6.0819	0.1087	0.0000	0.2298	0.0000	1.0098
RH	Dawn	OLS	Spherical	0.1528	1.3156	1.4684	5.4654	5.4654	0.1041	−0.0003	1.2314	−0.0001	1.0084
	Morning	OLS	Spherical	0.5867	3.9106	4.4973	5.1147	5.1147	0.1305	−0.0002	2.1239	−0.0001	0.9929
	Afternoon	OLS	Spherical	0.0000	9.4850	9.4850	5.0397	5.0397	0.0000	−0.0004	3.0743	−0.0001	0.9895
	Night	OLS	Spherical	0.3491	4.5540	4.9031	5.2721	5.2721	0.0712	−0.0004	2.2516	−0.0001	1.0085
THI	Dawn	OLS	Spherical	0.0003	0.1718	0.1721	5.8403	5.8403	0.0017	−0.0001	0.4225	−0.0001	1.0146
	Morning	OLS	Spherical	0.0000	0.2418	0.2418	5.0215	5.0215	0.0000	0.0000	0.4939	0.0000	0.9957
	Afternoon	OLS	Spherical	0.0002	0.1731	0.1733	5.8705	5.8706	0.0012	−0.0001	0.4892	−0.0001	1.1706
	Night	OLS	Spherical	0.0075	0.1565	0.1640	5.3975	5.3975	0.0457	0.0000	0.4047	0.0000	0.9917
*h*	Dawn	OLS	Spherical	0.0004	0.3014	0.3018	5.6248	5.6248	0.0013	−0.0002	0.5615	−0.0002	1.0160
	Morning	OLS	Exponential	0.0000	0.5309	0.5309	5.0008	14.9812	0.0000	−0.0056	0.6372	−0.0046	1.0423
	Afternoon	OLS	Spherical	0.0000	1.2355	1.2355	6.5328	6.5328	0.0000	−0.0033	1.1182	−0.0015	1.0121
	Night	OLS	Spherical	0.0209	0.5211	0.5420	5.4957	5.4957	0.0386	−0.0002	0.7542	−0.0001	1.0173

*C*_0_—nugget effect; *C*_1_—contribution; *C*_0_ + *C*_1_—sill; *a*—range; *a’*—practical range; SDI—spatial dependence index; ME—mean error; SD_M_—mean-error standard deviation; RE—reduced error; SDR—reduced-error standard deviation; OLS—ordinary least squares; REML—restricted maximum likelihood.

## Data Availability

The data presented in this study are available upon request from the corresponding author.
